# *In vitro* assessment of laser-ablated nanoparticles' cytotoxicity against different oral cancer cells

**DOI:** 10.6026/973206300212393

**Published:** 2025-08-31

**Authors:** Soumendu Bikash Maiti, Pratima Ngangom, Lalit Narayan Singh, Aaquib Nazir, Manjeet Kumar Shrivastava, Sukhpal Singh, Miral Mehta, Jugajyoti Pathi

**Affiliations:** 1 Department of Oral Medicine and Radiology, Government Dental College and Hospital, Jamnagar, Gujarat, India; 2 Department of Dental, CHC Kakching, Manipur Health Services, Manipur, India; 3 Department of Dentistry, Autonomous State Medical College, Sultanpur, Uttar Pradesh, India; 4 Department of Dentistry, SNM District Hospital, Leh, UT Ladakh, India; 5 Department of Oral Pathology and Microbiology, Divya Jyoti College of Dental Science and Research, Modinagar, Uttar Pradesh, India; 6 Department of Pediatric and Preventive Dentistry, Karnavati School of Dentistry, Karnavati University, Gandhinagar, Gujarat, India; 7 Department of Oral & Maxillofacial Surgery, Kalinga Institute of Dental Sciences, KIIT Deemed to be University, Bhubaneswar, Odisha, India

**Keywords:** Oral squamous cell carcinoma, gold nanoparticles, laser ablation, apoptosis, nanomedicinec

## Abstract

Oral cancer is an international health problem that has few treatment methods and a negative prognosis in late cases. The potential cytotoxicity of laser-ablated gold-silver and copper nanoparticles was considered in this study and the effects were tested on oral squamous cell carcinoma cell lines (SCC-4, CAL-27, KB-3-1) and normal human oral keratinocytes (HOK). The most cytotoxic was gold nanoparticles (IC 50: 38.9-45.1 0g/mL) with a marked selectivity (p < 0.001), causing apoptosis, as which flow cytometry and morphological analysis were confirmed. The statistical results obtained were moderate for silver and copper nanoparticles. These results present laser-ablated gold as a potential oral cancer anticancer agent that is specific.

## Background:

Oral cancer is mainly a carcinoma of the epithelial cells lining a cavity (squamous cells) of the mouth, synonymous with oral squamous cell carcinoma (OSSC), which is considered the sixth most prevalent cancer in the global population, accounting for 389,846 new cases in 2022 [1]. Although the morbidity associated with conventional treatment modalities such as surgery, chemotherapy and radiotherapy has improved, five-year survival rates are still very low at about 64.3% [2] because of diagnosis at an advanced stage, aggressive behavior of the tumor and a dearth of treatment options [3]. The traditional modalities of cancer therapy in the oral cavity tend to be characterized by harsh side-effects, emergence of resistance in the cancerous cells, as well as poor adherence by the patients [4]. Although chemotherapy is highly effective in eradicating fast-growing cancerous cells, it is not selective and damages the healthy cells massively, resulting in systemic toxicity and low quality of life [5]. Such shortcomings have led to the search for new therapeutic approaches that will preferentially kill cancer cells with the least damage to non-tumor cells. Nanotechnology has become one of the technological revolutions in cancer treatment, which provides new possibilities of effective cancer treatment with targeted drug administration in common, unprecedented possibilities of cancer therapy application and improvement [6, 7]. Metal nanoparticles, specifically, have aroused a lot of interest because of the special physical-chemical properties, non-toxicity and cell interaction characteristics [8, 9]. Laser ablation in a liquid medium, as one of the synthesis techniques, has been identified as an efficient and eco-friendly approach for generating pure and stable nanoparticles of a specific size and shape [10, 11]. Laser ablation synthesis has various advantages over traditional chemical techniques; these are the non-use of toxic reducing reagents, high particle purity and fine control of particle properties [12]. It takes the form of laser pulses of high energy and metallic targets of liquid medium that induce rapid heating, condensation, vaporization and the formation of ablated material into nanoparticles [13]. The method has so far been used with success to produce a variety of metal nanoparticles, such as gold, silver and copper nanoparticles, with prospective biomedical usages [14, 15]. The recent studies have revealed the anticancer capability of laser-ablated nanoparticles on the distinct lines of cancer cells [16, 17]. Cytotoxic impacts of these nanoparticles have been noted as a result of various mechanisms such as the creation of reactive oxygen species, cellular membrane disruption, obtrusion of cellular metabolism and provoking apoptosis [5, 18]. Nevertheless, even though the encouraging findings have been established with regard to other types of cancer, there is a lack of thorough investigations examining the cytotoxic effects of laser-ablated nanoparticles against specific cancer cells of the oral cavity.

## Materials and Methods:

The current experimental *In vitro* study aimed at assessing the cytotoxicity of laser-ablated nanoparticles on an oral cancer cell line. An experimental design based on a comparison framework composed the evaluation of the differential cytotoxicity of gold, silver and copper nanoparticles in the three cell lines of oral squamous cell carcinoma, as well as in normal human oral keratinocytes (HOK). Dynamic laser ablation synthesis in a liquid medium was used to synthesize nanoparticles. The gold, silver and copper metal targets were in high purity (99.99 */sup>2% (gold, silver and copper) medium and were placed in a water layer (double distilled). A Q-switched Nd:YAG laser source (wavelength of 1064 nm) was used to irradiate materials. The laser parameters obtained were an optimized pulse of 10 nanoseconds, 10 Hz repeating rate, 2.5 J/cm 2 fluence and an ablation time being 30 minutes. Colloidal nanoparticle suspensions obtained were stored at 4 ° C to be used again.

## Characterization and cell culture:

Transmission electron microscopy (TEM) and scanning electron microscopy (SEM) were used to characterize the synthesized nanoparticles in order to determine the size and shape of the particles. The crystalline nature of the samples was proven with the help of X-ray diffraction (XRD) and the surface plasmon resonance peaks were examined with the help of UV-visible spectroscopy. Hydrodynamic diameter and polydispersity index were determined by the use of dynamic light scattering (DLS). In the biological test, SCC-4, CAL-27 and KB-3-1, which are three human oral squamous cell carcinoma cell lines, were utilized. Non-cancerous controls were normal human oral keratinocytes (HOK). SCC-4 and CAL-27 were grown in DMEM, which contained 10% fetal bovine serum, as well as antibiotics, whereas KB-3-1 was kept in EMEM. Under the incubation conditions of oral keratinocyte medium and supplementation with a particular growth factor, HOK cells were cultured. All the cultures were incubated at 37 ° C and 5 percent CO 2 and sub cultured at 80-90 percent confluency.

## Treatment protocol and cytotoxicity assessment:

Each experimental condition included a minimum of six replicates to ensure statistical robustness. Cells were seeded at 5x10^3^ cells/well in 96-well plates for MTT assays and at 2x10^5^ cells/well in 6-well plates for microscopy and flow cytometry. After 24 hours of attachment, cells were treated with nanoparticles at concentrations ranging from 5 to 200 µg/mL for 24 and 48 hours. Control groups included untreated cells and vehicle controls. Cell viability was measured using the MTT assay. Post-treatment, MTT solution was added and incubated for 4 hours at 37°C. Formazan crystals were solubilized in DMSO and absorbance was read at 570 nm. Viability was calculated relative to untreated controls.

## Morphological and apoptosis analysis:

Morphological changes were monitored using phase-contrast microscopy at 6, 12, 24 and 48 hours post-treatment. Images were captured at 200x magnification to document features such as membrane blebbing, cell shrinkage and detachment. For apoptosis detection, annexin V-FITC/propidium iodide (PI) staining was performed following treatment with nanoparticle concentrations corresponding to their IC_50_ values. Flow cytometry was used to quantify early and late apoptotic as well as necrotic cell populations. Data were analyzed using appropriate software. All experiments were conducted in triplicate and repeated three times. Statistical analysis was performed using SPSS version 26.0. ANOVA with Tukey's post-hoc test and Student's t-test were used for group comparisons. IC_50_ values were determined through non-linear regression, with p-values <0.05 considered statistically significant.

## Results:

Laser ablation successfully produced gold, silver and copper nanoparticles with distinct physicochemical characteristics. Transmission electron microscopy (TEM) revealed spherical morphology for all three nanoparticle types. Gold nanoparticles displayed the smallest average diameter (18.3 ± 4.2 nm), followed by silver (22.7 ± 5.8 nm) and copper nanoparticles (28.4 ± 6.1 nm). UV-Visible spectroscopy confirmed stable colloidal formation with characteristic surface plasmon resonance peaks. Dynamic light scattering (DLS) results showed low polydispersity indices, indicating uniform size distribution (Table 1 - see PDF). All three types of nanoparticles demonstrated dose- and time-dependent cytotoxicity against oral squamous cell carcinoma cell lines (SCC-4, CAL-27, KB-3-1). Among them, gold nanoparticles showed the most potent cytotoxic effects, with IC_50_ values ranging from 38.9 to 45.1 µg/mL at 48 hours, whereas silver and copper nanoparticles were less effective, with higher IC_50_ values (Table 2 - see PDF). Notably, normal human oral keratinocytes (HOK) showed minimal cytotoxic response, with IC_50_ values above 150 µg/mL for all nanoparticles (p < 0.001), indicating good selectivity. Morphological analysis through phase-contrast microscopy confirmed progressive apoptotic features in treated cells, including membrane blebbing and cell shrinkage. Flow cytometry using annexin V/PI staining validated that apoptosis was the primary mechanism of cell death, with gold nanoparticles inducing apoptosis in over 67% of SCC-4 cells at 100 µg/mL (Table 3 - see PDF). Silver and copper nanoparticles induced comparatively lower levels of apoptosis. Necrosis remained under 10% in all treatment groups.

## Discussion:

The findings of this study demonstrate that laser-ablated nanoparticles, particularly gold nanoparticles, exhibit potent and selective cytotoxic effects against oral squamous cell carcinoma cells. These results are consistent with previous investigations that have reported the anticancer potential of metal nanoparticles synthesized through various methods [14, 15 and 17]. However, this study provides the first comprehensive evaluation of laser-ablated nanoparticles specifically against oral cancer cell lines, addressing a significant gap in the current literature. The superior cytotoxicity of gold nanoparticles observed in our study aligns with findings from stannic oxide nanoparticle studies on oral cancer cells, which demonstrated significant anti-proliferative effects [18]. The mechanism of action appears to involve multiple pathways, including oxidative stress induction, disruption of cellular metabolism and activation of apoptotic cascades [5, 19]. Our flow cytometry results confirming apoptosis as the primary mode of cell death are consistent with these mechanistic understandings. The selective cytotoxicity toward cancer cells compared to normal oral keratinocytes represents a crucial advantage for potential therapeutic applications [20]. This selectivity can be attributed to the differential metabolic characteristics and membrane properties between malignant and normal cells [21]. Cancer cells typically exhibit altered cellular metabolism, increased reactive oxygen species production and modified membrane compositions that may enhance their susceptibility to nanoparticle-induced damage [19]. The dose - and time-dependent cytotoxic effects observed in our study are consistent with previous reports on various nanoparticle systems [16, 22]. The IC_50_ values obtained for our laser-ablated nanoparticles fall within the range considered therapeutically relevant, with gold nanoparticles showing IC_50_ values comparable to those reported for conventional chemotherapeutic agents [23]. According to the National Cancer Institute classification, compounds with IC_50_ values below 20 µg/mL are considered highly cytotoxic, while those with IC_50_ values between 21-200 µg/mL are classified as moderately cytotoxic [23]. The morphological changes observed in our study, including cell shrinkage, membrane blebbing and nuclear condensation, are characteristic features of apoptotic cell death [24, 25]. These observations are supported by recent studies on metal-based nanomaterials that have demonstrated similar morphological alterations in cancer cells [26, 27]. The progressive nature of these changes, from subtle early alterations to extensive late-stage modifications, reflects the temporal dynamics of the apoptotic process. Among the strengths of the study, it is possible to highlight the use of more than one oral cancer cell line, the exhaustive characterization of nanoparticles and the analysis of cytotoxicity and selectivity parameters. The use of common protocols and statistical tests increases the reliability and reproducibility of the results. Moreover, this study of several time points and concentrations focuses on great values of dose-responses when it comes to possible therapeutic applications. Nevertheless, one should take into consideration some limitations. The experiment was *In vitro* only and its findings cannot be representative of this *in vivo* tumor complex microenvironment. Behaviors of nanoparticles with serum proteins, immune cells and extracellular matrices may have strong impacts on their availability and effectiveness in clinical practice. Moreover, this study did not assess the long-term effects of this approach, as well as the possibility of developing resistance. The research directions towards the future should consider a three-dimensional cell culture model that is a more accurate mimic of the tumor microenvironment, combination therapy with conventional therapy and in-depth mechanistic studies to understand the exact molecular mechanisms involved in nanoparticle-induced cytotoxicity. Also, *in vivo* experiments with suitable animal models should be necessary to prove the therapeutic properties and safety of laser-ablated nanoparticles.

## Conclusion:

Selective cytotoxicity of laser-ablated gold nanoparticles towards oral squamous cell carcinoma with IC50 values of 38.9-45.1 microgram per millilitre and selectivity indices of 3.7-4.3. The type of cell death was apoptosis, which was confirmed by morphological changes and also through flow cytometry. The laser ablation-based green synthesis highlights its potential to have sustainable cancer treatment using nanomedicine.

## References

[1] Bellantoni MI (2023). Biomedicines..

[2] HanY et (2014). Int J Nanomedicine..

[3] Kim M (2017). KONA Powder Part J..

[4] Abid S (2020). J Mater Res Technol..

[5] Mahadevaiah (2019). J Biosci..

[6] Li H (2020). Front Bioeng Biotechnol..

[7] Lee G, Park YII. (2018). Nanomaterials..

[8] Mahesh KP (2023). J Indian Acad Oral Med Radiol..

[9] Hum NR (2020). Cancers (Basel)..

[10] Meerloo JV (2011). Methods Mol Biol..

[11] Riss TL (2016). Assay Guidance Manual..

[12] Sargent JM. (2003). Recent Results Cancer Res..

[13] Fazio E (2020). Nanomaterials (Basel)..

[14] Zein-Sabatto A (2025). Sci Rep..

[15] Chauncey R (2018). Curr Res Dent..

[16] Afrasiabi M (2022). Iran J Pharm Res..

[17] Tang XH (2004). Clin Cancer Res..

[18] Saghiri MA (2025). Cancer Control..

[19] Maghimaa M (2025). Inorganic Chemistry Communications..

[20] https://www.wcrf.org/.

[21] Conway DI (2008). Int J Cancer..

[22] https://www.nidcr.nih.gov/.

[23] Correard F (2014). Int J Nanomedicine..

[24] Luobin L (2024). Cell Death Dis..

[25] Hannun YA. (1997). Blood..

[26] Huo M (2017). Nat Commun..

[27] Mahesh KP (2023). Journal of Indian Academy of Oral Medicine & Radiology..

